# Successful outcomes using Long Acting Buprenorphine (LAB - Buvidal) to treat Codeine, Tramadol and other Opioid Analgesia Dependencies (OAD) in Wales during the Pandemic

**DOI:** 10.1192/j.eurpsy.2022.855

**Published:** 2022-09-01

**Authors:** J. Melichar, F. Graver, L. Parimelalagan, J. Lewis

**Affiliations:** 1 University of South Wales, Health, Sport & Professonal Practice, Cardiff, United Kingdom; 2 Bath University, Pharmacy & Pharmacology, Bath, United Kingdom; 3 Cardiff and Vale University Health Board, Community Addiction Unit, Cardiff, United Kingdom; 4 Betsi Cadwaladr University Health Board, Community Drugs And Alcohol Services, Rhyl, United Kingdom; 5 Aneurin Bevan University Health Board, Community Drugs And Alcohol Services, Newport, United Kingdom

**Keywords:** buprenorphine, buvidal, pain, long acting

## Abstract

**Introduction:**

Long Acting Buprenorphine (LAB) – Buvidal (CAM2038) – is a prolonged release treatment for opioid dependence in adults. Its extensive use was funded by Welsh Government during the pandemic in Wales and it has been found to be a significantly better than oral medications in improving quality of life, possibly through providing allostatic craving and anxiety reduction

**Objectives:**

This is a case series of 10 patients who were referred to Community Addiction Services in North and South West Wales with OAD.

**Methods:**

Patients were mainly using Codeine or Tramadol and were referred due to either ongoing illicit use or via primary care services requesting support. As part of the pandemic initiative, they were initiated on Buvidal and followed up.

**Results:**

All ten patients successfully started on Buvidal without significant issues. As a group, if transferred straight to Buvidal, they tended to have fewer significant withdrawal symptoms prior to starting on the Buvidal compared to those on Methadone or Heroin. They were treated on the usual range of Buvidal doses (1 on 64mg, the others on 96-128mg monthly). They have all stabilised and successfully moved on with their lives on Buvidal. One has used the time on Buvidal to have psychological input around past traumas and successfully detoxified in the community using Buvidal.

**Conclusions:**

Recommendations for services considering OAD - it is a surprisingly effective treatment which is easy to start. It has the scope for being both an effective OAD recovery medication and a potentially simple detoxification strategy for this patient group.

**Disclosure:**

Professor Melichar has provided consultancy work, presentations, training and chaired panel discussions for all the companies in this area in the UK and some outside the UK. Recent work includes Althea (UK), Britannia (UK), Camurus (UK and Global), Martin

